# Reformulation initiative for partial replacement of saturated with unsaturated fats in dairy foods attenuates the increase in LDL cholesterol and improves flow-mediated dilatation compared with conventional dairy: the randomized, controlled REplacement of SaturatEd fat in dairy on Total cholesterol (RESET) study

**DOI:** 10.1093/ajcn/nqz344

**Published:** 2020-02-05

**Authors:** Dafni Vasilopoulou, Oonagh Markey, Kirsty E Kliem, Colette C Fagan, Alistair S Grandison, David J Humphries, Susan Todd, Kim G Jackson, David I Givens, Julie A Lovegrove

**Affiliations:** 1 Hugh Sinclair Unit of Human Nutrition and Institute for Cardiovascular and Metabolic Research, University of Reading, Reading, United Kingdom; 2 Animal, Dairy and Food Chain Sciences, University of Reading, Reading, United Kingdom; 3 Institute for Food, Nutrition and Health, University of Reading, Reading, United Kingdom; 4 Department of Mathematics and Statistics, University of Reading, Reading, United Kingdom

**Keywords:** dairy fat, cardiovascular disease risk, cholesterol profile, flow-mediated dilatation, saturated fatty acids, monounsaturated fatty acids, vascular function, reformulation, food chain approach

## Abstract

**Background:**

Modifying dairy fat composition by increasing the MUFA content is a potential strategy to reduce dietary SFA intake for cardiovascular disease (CVD) prevention in the population.

**Objectives:**

To determine the effects of consuming SFA-reduced, MUFA-enriched (modified) dairy products, compared with conventional dairy products (control), on the fasting cholesterol profile (primary outcome), endothelial function assessed by flow-mediated dilatation (FMD; key secondary outcome), and other cardiometabolic risk markers.

**Methods:**

A double-blind, randomized, controlled crossover 12-wk intervention was conducted. Participants with a 1.5-fold higher (moderate) CVD risk than the population mean replaced habitual dairy products with study products (milk, cheese, and butter) to achieve a high-fat, high-dairy isoenergetic daily dietary exchange [38% of total energy intake (%TE) from fat: control (dietary target: 19%TE SFA; 11%TE MUFA) and modified (16%TE SFA; 14%TE MUFA) diet].

**Results:**

Fifty-four participants (57.4% men; mean ± SEM age: 52 ± 3 y; BMI: 25.8 ± 0.5 kg/m^2^) completed the study. The modified diet attenuated the rise in fasting LDL cholesterol observed with the control diet (0.03 ± 0.06 mmol/L and 0.19 ± 0.05 mmol/L, respectively; *P *= 0.03). Relative to baseline, the %FMD response increased after the modified diet (0.35% ± 0.15%), whereas a decrease was observed after the control diet (−0.51% ± 0.15%; *P*< 0.0001). In addition, fasting plasma nitrite concentrations increased after the modified diet, yet decreased after the control diet (0.02 ± 0.01 μmol/L and −0.03 ± 0.02 μmol/L, respectively; *P *= 0.01).

**Conclusions:**

In adults at moderate CVD risk, consumption of a high-fat diet containing SFA-reduced, MUFA-enriched dairy products for 12 wk showed beneficial effects on fasting LDL cholesterol and endothelial function compared with conventional dairy products. Our findings indicate that fatty acid modification of dairy products may have potential as a public health strategy aimed at CVD risk reduction. This trial was registered at clinicaltrials.gov as NCT02089035.

## Introduction

High intakes of SFAs have been associated with an increase in LDL cholesterol concentrations (1), an established risk factor in the development of cardiovascular diseases (CVDs) (2). Although dairy products contribute ≤27% of total dietary SFA intake in the UK (3), the majority of prospective studies and meta-analyses show either a neutral or reduced risk of CVD with increased consumption of milk and dairy products, with the exception of butter (4). This finding may reflect the complexity of the dairy food matrix (4), comprised of a mixture of macronutrients, micronutrients, and other components (e.g., bioactive peptides), with studies indicating that consuming dairy fat as hard cheese may provide beneficial blood lipid effects compared with butter (5, 6).

Some meta-analyses have questioned the link between high SFA intake and CVD risk and mortality (7, 8); however, evidence indicates replacement of SFAs with unsaturated fatty acids (FAs) is beneficial (9, 10). Interventional and observational data have shown that substitution of dietary SFAs with MUFAs (11) or PUFAs (11–14) is linked to reductions in CVD risk biomarkers and events. Partial replacement of SFAs in milk fat with unsaturated FAs, primarily in the form of *cis*-MUFAs, can be achieved by supplementing the dairy cow diet with plant oils or oil seeds (15). This innovative animal nutrition–based reformulation strategy could limit the entry of SFAs into the food chain, without removing the beneficial aspects of dairy product consumption (16, 17). Although evidence is insufficient to draw firm conclusions, FA-modified dairy foods (primarily butter) have the potential to beneficially affect established cholesterol-based markers of CVD risk, relative to conventional dairy products (18).

Since dairy products are complex in nature and CVD is multifactorial, there is an urgent need to consider both established lipid biomarkers and novel risk factors, including endothelial function, arterial stiffness, and inflammatory markers, when investigating the impact of FA-modified dairy food consumption (16). In particular, measures of endothelial function provide independent prognostic data on future cardiovascular events and flow-mediated dilatation (FMD) is regarded as the gold-standard technique for the assessment of endothelial-dependent vasodilation and a surrogate measure of endothelial NO production (19, 20).

To address this knowledge gap, we investigated the impact of a high-fat, high-dairy diet incorporating SFA-reduced, MUFA-enriched dairy products, modified via a food chain approach, on established and novel CVD risk markers, compared with dairy foods with an FA profile typical of conventional retail products (control), in adults at moderate CVD risk. In our proof-of-concept study, we hypothesized that consumption of the modified dairy products for 12 wk would improve fasting total cholesterol (TC; composed of LDL cholesterol and HDL cholesterol; primary outcome), the %FMD response (key secondary outcome), and other cardiometabolic risk markers (secondary outcomes), compared with consumption of the control products.

## Methods

### Subjects and study design

The REplacement of SaturatEd fat in dairy on Total cholesterol (RESET) study (NCT02089035) was given a favorable ethical opinion for conduct by the University of Reading Research Ethics Committee (Ref. No.: 13/43) and carried out in accordance with the principles of the Declaration of Helsinki of 1975, as revised in 1983. Men and women (aged 25–70 y; BMI: 19–32 kg/m^2^) with moderate CVD risk were recruited from Berkshire, United Kingdom, from February 2014 to September 2015 in 3 cohorts. To assist with participant recruitment, the study age range was extended to 70 y from May 2014. The study was completed in April 2016. All participants provided written informed consent. Details of the study inclusion and exclusion criteria are reported in Markey et al. (21). In summary, eligibility criteria included no diagnosis of myocardial infarction, stroke, or diabetes; not taking dietary supplements or medication for hyperlipidemia, hypertension, hypercoagulation, or inflammatory conditions; normal biochemistry for liver and kidney function; not pregnant or lactating; not participating in excessive amounts of vigorous aerobic physical activity (<3 times × 20 min/wk); and not consuming excessive amounts of alcohol (men: <21 units/wk; women: <14 units/wk). A total of 130 potential participants attended a screening visit, resulting in 76 eligible for random assignment (see **Supplemental Figure 1** for the participant flowchart). Data collected at the screening visit on fasting serum TC, HDL cholesterol, glucose, clinic blood pressure (BP), BMI, and family history of myocardial infarction were used to determine eligibility, whereby a score of ≥2 CVD risk points based on a modified Framingham risk analysis suggested a 1.5-fold higher (moderate) CVD risk than the population mean (11, 21, 22).

The RESET intervention was a double-blind, crossover, randomized controlled proof-of-concept study with an 8-wk washout period between the two 12-wk high-fat, high-dairy dietary periods. A minimization technique stratifying by information on age (25.0–40.0 y, 40.1–55.1 y, or 55.2–70.0 y), gender (male or female), BMI (19.0–26.9 or 27.0–32.0 kg/m^2^), and fasting serum TC (≤5.9 or ≥6.0–8.0 mmol/L) was used by a single researcher (OM) to randomly assign eligible participants to 1 of 2 dietary intervention groups, whereby Group 1 received the modified diet first (Diet A) followed by the control diet (Diet B), and vice versa for Group 2 (21). This randomization approach was employed to minimize the imbalance of potential confounding variables between groups with a different order of intervention diet. Data processing and analysis of samples were completed in a blinded fashion; biochemical analysis and raw data were labeled by period number (i.e., visit 1, 2, 3, or 4), not by intervention diet.

### Intervention diets

Full details of the production of the SFA-reduced, MUFA-enriched (modified) milk and dairy products have been published elsewhere (15). Briefly, after a 4-wk supplementation of the total mixed ration diet of multiparous Holstein-Friesian dairy cows with ∼1 kg high-oleic sunflower oil per cow per day (AAK Ltd.), the milk was collected for the manufacture of ultra-high temperature (UHT) milk, cheddar cheese, and butter. Control dairy products, with an FA profile typical of those available in supermarkets, were provided by Arla Foods UK. To ensure that participants and investigators remained blinded to the diets, an investigator (CCF) not involved in running the dietary intervention was responsible for ensuring that dairy products were provided to participants in plain packaging and were only identifiable by a single-letter code (A or B).

A food-exchange model was designed for the RESET study, based on the strategy for adults with moderate CVD risk described in detail in Markey et al. (21). Participants were provided with the study products, in blinded packaging, to take home and were asked to consume 340 g/d of UHT milk, 45 g/d of cheddar cheese, and 21.5 g/d (control diet) or 25.1 g/d (modified diet) of butter. **Table 1** presents the daily energy and nutrient intakes provided by the study products. As described previously (21), participants received individual verbal and written instructions to incorporate the study products into their habitual diet after the baseline clinical visit (week 0). The isoenergetic high-fat, high-dairy daily dietary exchange [38% of total energy intake (%TE) from total fat] was achieved by replacing dairy products, oil/spreads, and snacks habitually consumed with either the SFA-reduced, MUFA-enriched (modified) UHT milk, cheddar cheese, and butter (dietary target: 16%TE SFA; 14%TE MUFA) or matched, conventional dairy foods (control) with an FA profile typical of retail products (19%TE SFA; 11%TE MUFA). The 2 intervention diets were equal in total exchangeable fat content (∼41 g/d of dairy fat).

**TABLE 1:** Daily energy and nutrient intakes provided by the study products in the modified and control intervention diets^1^

	Modified				Control			
	UHT milk	Cheese	Butter	Total	UHT milk	Cheese	Butter	Total
Daily amount, g	340.0	45.0	25.1	—	340.0	45.0	21.5	—
Energy, MJ/d	0.79	0.69	0.76	2.24	0.75	0.76	0.65	2.16
Total fat, g/d	8.8	12.6	20.4	41.8	8.6	15.1	17.4	41.1
SFAs, g/d	4.4	6.3	10.2	20.9	5.7	10.2	11.1	27.0
MUFAs, g/d	3.6	5.0	8.1	16.7	2.1	3.5	4.4	10.0
PUFAs, g/d	0.3	0.4	0.7	1.4	0.3	0.4	0.6	1.3
TFAs, g/d	0.9	1.4	2.1	4.4	0.3	0.5	0.7	1.5
CHO, g/d	16.0	1.2	0.2	17.4	14.9	1.4	0.4	16.7
Protein, g/d	11.1	12.1	0.1	23.3	10.2	10.6	0	20.8
Calcium, mg/d	404.0	409.8	4.5	818.3	428.0	340.5	3.7	772.2
Magnesium, mg/d	37.4	13.6	0.5	51.5	39.0	13.5	0.4	52.9
Sodium, mg/d	154.8	341.9	125.9	622.6	140.7	326.5	152.1	619.3
Phosphorus, mg/d	303.3	269.5	5.9	578.7	333.5	242.4	4.9	580.8

1 Adapted from Kliem et al. (15) and Markey et al. (21). Values represent daily required intakes of the 3 study products. CHO, carbohydrates; TFA, *trans* fatty acid; UHT, ultra-high temperature.

Compliance was assessed using 4-d weighed food diet diaries (weeks 0, 12, 20, and 32), daily records of study product consumption, and analysis of plasma phospholipid FAs (PL-FAs) which served as a short-term biomarker of dietary fat composition. To maintain a constant body weight, participants were weighed every 4 wk by the investigators and any changes ≥±1 kg were addressed. At baseline, our cohort had a mean habitual total fat intake of ∼36%TE (see **Supplemental Table 1**for reported dietary intake), which was higher than the total fat intake estimated for the UK adult (19–64 y) population, at 33.2%TE (3), but is comparable with total fat intake in adults at moderate CVD risk [see the protocol article for details (21)]. Relative to the control diet, consumption of the modified diet led to a mean decrease of SFA intake of 2.5%TE and a parallel mean increase of MUFA intake of 3.7%TE (Supplemental Table 1) (21). Results from the analysis of the PL-FA composition, which showed a significant difference between the modified and control treatments after the two 12-wk interventions (*P *≤ 0.01), were previously presented (for a summary of the results, see **Supplemental Table 2**) (21). We previously showed that consumers generally accepted our FA-modified milk and dairy products, when tasted in a blinded manner (23).

In addition, self-reported physical activity was assessed using the self-administered, last 7-d version of the International Physical Activity Questionnaire (IPAQ)-long form. A 100-mm visual analog scale (VAS) questionnaire was also completed by participants at the end of each 12-wk intervention period to assess the visual appeal, smell, taste, aftertaste, and palatability of the UHT milk, cheese, and butter.

### Study visits

Study visits were conducted in a temperature-controlled environment (22 ± 1°C) at the Hugh Sinclair Unit of Human Nutrition, University of Reading, during weeks 0, 12, 20, and 32. Premenopausal women attended all visits during the same phase of their menstrual cycles. Participants were asked to refrain from alcohol and aerobic exercise for 24 h before each visit and to fast overnight for 12 h after consuming a low-fat standard meal (<1.46 MJ; <7 g total fat) that was provided by the researchers. Participants drank only low-nitrate mineral water (Buxton Mineral Water, Nestlé Waters UK) during the 12-h fast and on the morning of the study visit. After a 30-min rest in the supine position, the same trained researcher performed noninvasive vascular function measurements at all 4 visits for each study participant. Fasted venous blood samples were then collected into lithium heparin–coated, EDTA-coated, or serum separator blood collection tubes (VACUETTE, Greiner Bio-One) and either kept briefly on ice (plasma samples) or left to clot for ≥30 min at room temperature (serum samples) before centrifugation at 1700 × *g* for 15 min at 4°C. Plasma and serum were divided into aliquots before storage at −80°C until analysis.

### Biochemical analyses, risk scores, and predictive equations

Serum lipids [TC, HDL cholesterol, and triacylglycerol (TAG)], glucose, apoB, C-reactive protein, and nonesterified fatty acids (NEFAs) were measured using an ILAB 600 autoanalyzer (TC, HDL cholesterol, TAG, glucose, and ultrasensitive C-reactive protein reagents and autoanalyzer: Werfen UK Ltd.; apoB reagent: Randox Laboratories Ltd.; NEFA reagent: Alpha Laboratories Ltd.). The Friedewald equation was used to estimate fasting LDL cholesterol (24). ELISA kits were used to analyze serum insulin (Dako UK Ltd.), plasma vascular adhesion molecule 1 (VCAM-1) and intercellular adhesion molecule 1 (ICAM-1), E-selectin, and P-selectin (R&D Systems Europe Ltd.). Insulin resistance was estimated by HOMA-IR, whereas insulin sensitivity was estimated with the revised quantitative insulin sensitivity index (rQUICKI), using standard equations (25). Measurement of plasma nitrite and nitrate was determined by HPLC (ENO-30, EiCom Corporation) coupled with online reduction of nitrate to nitrite, and postcolumn derivatization with the Greiss reagent (ENO-30 Analyzer, EiCom Corporation) (26). Mean interassay CVs were <5% for automated assays and <10% for all other assays.

Risk scores pre- and postintervention for both diets were calculated using the modified Framingham CVD score (11, 21). Furthermore, postintervention changes in dietary total FA intake, as assessed by the 4-d weighed food diet diaries, were used to calculate the predicted change in TC and LDL cholesterol with the use of 5 published equations (27), which included change in FA intake of total SFAs, MUFAs, and PUFAs as summarized in **Supplemental Table 3**.

### NMR metabolomics

A high-throughput ^1^H-NMR metabolomics platform (Nightingale Health Ltd.) was used to quantify the particle size of the subclasses of LDL and HDL, and concentrations of particles within each subclass, as previously described (28). This article will present the change-from-baseline (Δ) after the 2 intervention diets for the LDL [classified by their mean diameter size as small (18.7 nm), medium (23 nm), and large (25.5 nm)] and HDL [classified as small (8.7 nm), medium (10.9 nm), large (12.1 nm), and very large (14.3 nm)] concentrations according to particle size (28).

### Assessment of vascular outcomes and 24-h ambulatory BP

Before assessing the vascular function measurements, clinic supine BP was measured on the left upper arm with the use of a validated BP monitor (Omron MX2 Digital Automatic Upper Arm Blood Pressure Monitor; OMRON). Endothelial function was assessed using FMD as described elsewhere (29). Briefly, trained researchers used a CX50 CompactXtreme portable ultrasound system (Philips HealthCare) to determine endothelial-dependent vasodilation of the brachial artery following defined guidelines. Electrocardiogram-gated images were captured at 0.25 frames/s for 660 s with the use of image-grabbing software (Vascular Research Tools 5; Medical Imaging Applications LLC) (29). The analysis of obtained images was performed in a blinded manner, with the use of wall-tracking software (Vascular Research Tools 5; Medical Imaging Applications LLC). The %FMD response was calculated as (diameter_max _− diameter_baseline_)/diameter_baseline _× 100. For each image, the %FMD response was assessed in triplicate, from which the mean response was calculated. Reduced bioavailability of NO plays a significant role in endothelial dysfunction, as indicated by an impaired brachial artery %FMD response (19). Preocclusion and peak artery diameter and time to peak diameter were also determined.

The carotid intima media thickness (cIMT), defined as the distance between the media–adventitia interface and the intima–lumen interface, was assessed using a 12-3 linear array transducer and CX50 ultrasound system (Philips HealthCare) on the right and left common carotid arteries, following published guidelines (30). Using automated edge-tracking software (QLAB, Philips), images of the far-wall cIMT were captured when the carotid vessel was at its widest diameter, reflecting artery expansion. The mean of both the right and left sides was used to represent the cIMT value (30).

Radial pulse wave analysis (PWA) and carotid-femoral pulse wave velocity (PWV; m/s) were measured in triplicate using a SphygmoCor (AtCor Medical), as previously described (11). PWA determined the augmentation index, which was corrected for a heart rate of 75 beats/min (%). The stiffness index (m/s) and reflection index (%) were determined by digital volume pulse (DVP; Pulse Trace PCA2, Micro Medical Ltd.), providing measures of arterial stiffness and vascular tone, respectively (11).

Ambulatory blood pressure (ABP) and heart rate were measured using A/A grade automated oscillometric ABP monitors (A&D Instruments Ltd.) every 30 min from 07:00 to 21:59 and every 60 min from 22:00 to 06:59 (11). Participants were asked to wear the ABP monitors for ≥48 h before each of the 4 visits and repeat each measurement on the same selected day of the week. Sleep times were recorded by the participants and used to estimate mean 24-h day and night measurements. Pulse pressure was calculated as the difference between systolic BP and diastolic BP.

### Statistical analysis

For our primary outcome (serum TC, composed of LDL cholesterol and HDL cholesterol), a total of 54 eligible participants were required to achieve a ∼0.3 mmol/L predicted reduction in TC (using the Keys equation) taking into consideration a difference of 4.0%TE from SFAs and a population mean ± SD of 4.54 ± 0.5 mmol/L, 80% power, 5% significance level, and allowing for a 15% dropout rate (31). Accounting for dropout, 46 participants were needed to complete the study. The same cohort size was also sufficient to detect a 1.5% intergroup difference in the %FMD response (key secondary outcome), with a power of 80% at *P* < 0.05 (32). A*P* value of <0.05 was considered significant for the primary (TC) and the key secondary (%FMD response) outcomes. Other secondary outcome measures were fasting lipid profile (TAG, apoB, and NEFA); HDL/LDL particle size distribution; indexes of insulin resistance (glucose, insulin, HOMA-IR, rQUICKI); measurements of arterial stiffness (PWA, PWV, DVP) and cIMT; clinic BP; 24-h ABP; circulating biomarkers of endothelial activation and inflammation (nitrite, nitrate, VCAM-1, ICAM-1, E-selectin, P-selectin, C-reactive protein); and CVD risk scores. For the other secondary outcome variables, no formal sample size calculations were performed. A *P* value of ≤0.01 was chosen a priori when assessing significance for these variables to acknowledge the issue of multiple comparisons but also to allow identification of interesting findings (33). Results in tables are presented as unadjusted means ± SEMs.

Statistical analyses were predominantly conducted using the SAS 9.4 University edition statistical software (SAS Institute Inc.). All variables were checked for normality and data were logarithmically transformed where needed. Treatment effects were evaluated for all primary and secondary outcomes using a linear mixed model, with Δ for each 12-wk dietary intervention (calculated by subtracting week 0 from week 12 values; and week 20 from week 32 values) as the dependent variable, adjusted for fixed effects of baseline values of the assessed variable, period, sequence, treatment, age, gender, and BMI. Fixed-effect covariates were retained in the linear mixed model irrespective of their degrees of significance. Participant was included as a random effect. No period effects were observed in the model for any outcome measure. Results from the VAS questionnaire were analyzed using paired *t* tests to assess differences between study product ratings. Baseline characteristics of participants randomly assigned to consume the modified and control dairy products during their first dietary exchange period (i.e., week 0) were assessed by independent *t* tests and chi-square tests for continuous and categorical variables, respectively. Since this is a proof-of-concept study rather than a confirmatory trial, we chose to adopt a per-protocol analysis approach a priori (21). This approach was taken, rather than intention-to-treat, because the study is designed to evaluate efficacy rather than effectiveness (34).

## Results

### Study participants, CVD risk score, and product rating

A total of 76 eligible participants were randomly recruited to the intervention, 54 of whom completed both arms of the study successfully (Supplemental Figure 1). Reasons for dropout included: unable to comply with the intervention (*n* = 9); time commitment (*n* = 8); and health or personal issues unrelated to the intervention (*n* = 5). While undergoing the modified or the control dietary exchange period, 7 participants withdrew from each arm; this suggests that the diets were similar in terms of acceptability (21). **Table 2** shows baseline characteristics (at the start of the first dietary intervention period) of the 54 participants who completed the study. As outlined previously (21), there were no significant differences in the baseline characteristics of participants randomly allocated to the modified and control dietary interventions at baseline (week 0). Inclusion of dropouts did not affect baseline demographic characteristics.

**TABLE 2:** Characteristics of study participants at the beginning of the first dietary exchange period^1^

Characteristics	Overall group (*n* = 54)	Modified (*n* = 24)	Control (*n* = 30)
Age, y	52 ± 3	51 ± 3	52 ± 2
Sex			
Men	31 (57.4)	15 (62.5)	16 (53.3)
Women	23 (42.6)	9 (37.5)	14 (46.7)
BMI, kg/m^2^	25.8 ± 0.5	25.8 ± 0.7	25.0 ± 0.6
Waist circumference, cm	89.8 ± 1.4	90.2 ± 2.2	86.7 ± 1.9
Clinic SBP, mm Hg	123 ± 2	122 ± 2	119 ± 2
Clinic DBP, mm Hg	74 ± 1	74 ± 2	71 ± 1
Fasting serum biomarkers			
TC, mmol/L	5.49 ± 0.12	5.45 ± 0.18	5.60 ± 0.20
LDL-C, mmol/L	3.47 ± 0.11	3.40 ± 0.15	3.52 ± 0.15
HDL-C, mmol/L	1.48 ± 0.04	1.49 ± 0.08	1.46 ± 0.04
Triacylglycerol, mmol/L	1.17 ± 0.06	1.22 ± 0.11	1.14 ± 0.07
Glucose, mmol/L	5.37 ± 0.11	5.46 ± 0.22	5.37 ± 0.17
Insulin, pmol/L	36.3 ± 2.8	35.3 ± 3.7	37.1 ± 4.0
HOMA-IR	1.44 ± 0.11	1.41 ± 0.15	1.46 ± 0.16
CVD risk score^2^	3.0 ± 0.2	2.8 ± 0.2	3.2 ± 0.3

1 Adapted from Markey et al. (21). Values are mean ± SEM or *n* (%). *n *= 54 (overall group). No significant differences between groups of participants randomly assigned to consume the modified and control dairy products during their first dietary exchange period were identified for any of the baseline characteristics [independent *t* tests (continuous variables); chi-square test (categorical variable)]; *P *> 0.05. CVD, cardiovascular disease; DBP, diastolic blood pressure; HDL-C, HDL cholesterol; LDL-C, LDL cholesterol; SBP, systolic blood pressure; TC, total cholesterol.

2 Determined by using the REplacement of SaturatEd fat in dairy on Total cholesterol study screening tool (21). A score of ≥2 points relates to a 50% greater risk of CVD than the population mean.

Participant physical activity scores, based on the IPAQ questionnaire, did not significantly differ between treatments, relative to baseline (data not shown). The VAS questionnaire, which assessed the visual appeal, smell, taste, aftertaste, and palatability of the 3 dairy products after the modified and control diets, showed no significant treatment effect (data not shown).

### Fasting serum lipids and biochemical analyses

**FIGURE 1:**
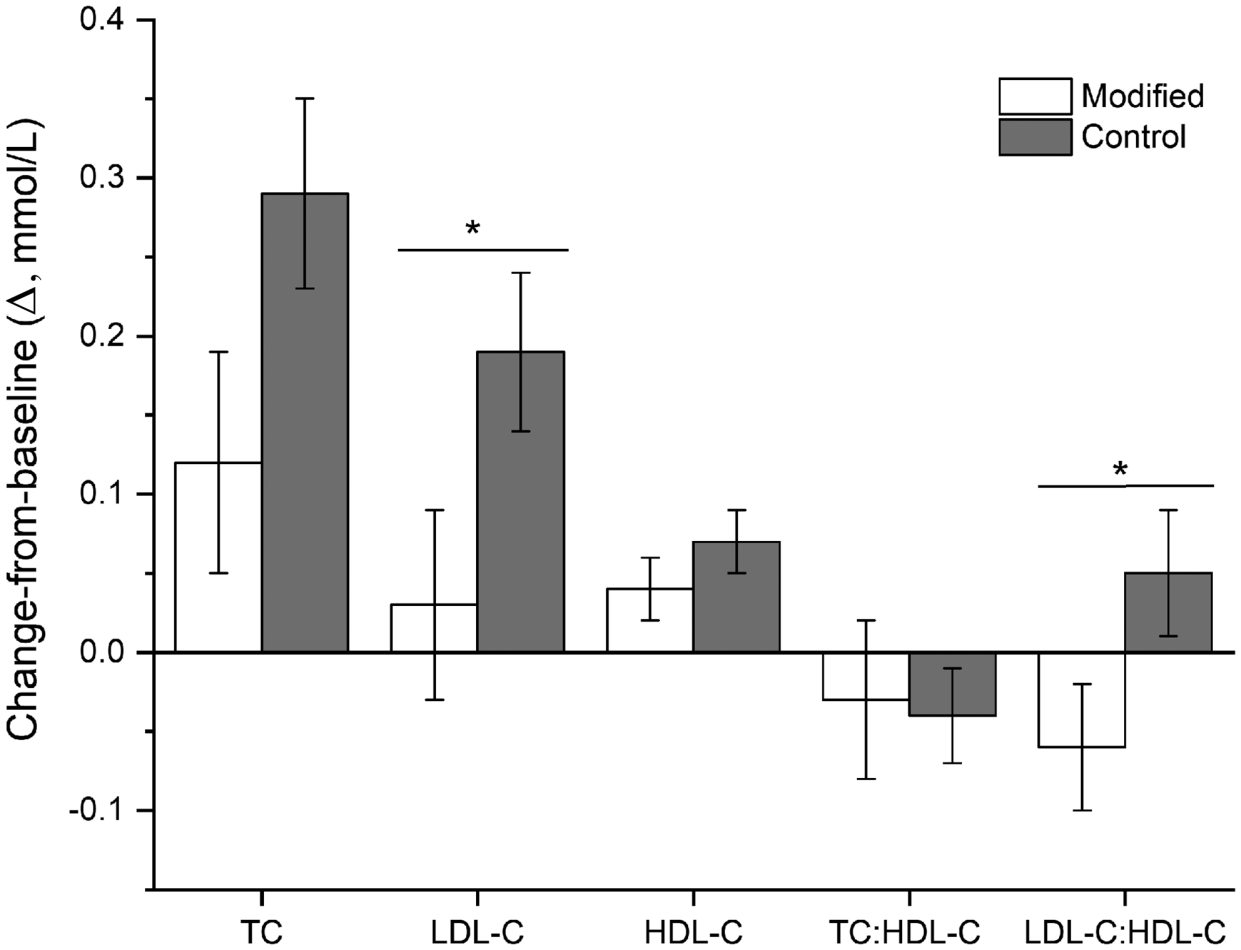
Δ fasting cholesterol profile after 12-wk diets that incorporated modified and control dairy products. Values are unadjusted means ± SEMs. *n = *54. Linear mixed-model analyses were used to calculate overall effect of treatment based on Δ values, with adjustments made for fixed effects of baseline values of the assessed variable, period, treatment sequence, gender, age, and BMI. Participant was included as a random effect. The Δ values after each 12-wk dietary intervention were calculated by subtracting week 0 from week 12 values; and week 20 from week 32 values. No period effects were observed in the model for any outcome measure. *P < 0.05. HDL-C, HDL cholesterol; LDL-C, LDL cholesterol; TC, total cholesterol; Δ, change-from-baseline.

Although there was no significant Δ in serum TC between diets (*P *= 0.08), consumption of the modified diet had a significant beneficial effect in terms of attenuation of the rise of the LDL cholesterol concentration after the control diet (*P *= 0.03; **Figure 1**, **Supplemental Table 4**). There was no evidence of a difference in Δ in HDL cholesterol between the 2 diets. As shown in Figure 1, there was a differential effect of the diets on the LDL cholesterol:HDL cholesterol ratio, which decreased significantly after the modified diet, whereas it increased after the control diet (*P *= 0.04). No significant treatment effect was observed for any other component of the lipid profile measured or indexes of insulin sensitivity/resistance (**Table 3**). There was a differential effect of the dairy fat diets on fasting plasma nitrite concentrations, with an increase observed relative to baseline after the modified diet and a decrease observed after the control diet (*P *= 0.01; **Table 4**). There was no significant effect of the diets on plasma nitrate or any of the markers of endothelial activation or inflammation (VCAM-1, ICAM-1, E-selectin, P-selectin, C-reactive protein; Table 4). Lastly, there was no differential effect of treatment on the CVD risk score, relative to baseline (data not shown).

**TABLE 3:** Fasting lipid profile, LDL/HDL particle size distributions, and indexes of insulin resistance at baseline (week 0/week 20) and postintervention (week 12/week 32), and the Δ after consumption of the modified and control diets^1^

	Modified			Control			
	Baseline	Post	Δ	Baseline	Post	Δ	*P* *^2^*
Fasting lipid profile							
Triacylglycerol, mmol/L	1.24 ± 0.07	1.35 ± 0.10	0.11 ± 0.07	1.18 ± 0.06	1.24 ± 0.07	0.06 ± 0.05	0.32
ApoB, g/L	1.01 ± 0.03	1.03 ± 0.03	0.02 ± 0.01	1.00 ± 0.03	1.03 ± 0.03	0.03 ± 0.01	0.47
NEFAs, μmol/L	567 ± 24	514 ± 23	−53 ± 27	556 ± 27	515 ± 23	−41 ± 27	0.84
Particle size distributions							
LDL,^3^ nmol/L							
Small	172 ± 4	175 ± 5	3 ± 1	169 ± 4	176 ± 4	7 ± 2	0.36
Medium	278 ± 8	282 ± 8	5 ± 2	268 ± 9	284 ± 8	16 ± 8	0.34
Large	570 ± 16	581 ± 17	11 ± 5	551 ± 18	581 ± 16	38 ± 19	0.34
HDL,^4^ nmol/L							
Small	10,100 ± 109	10,307 ± 128	207 ± 102	10,080 ± 125	10,354 ± 117	247 ± 112	0.85
Medium	3974 ± 102	4106 ± 111	132 ± 52	4028 ± 111	4118 ± 117	87 ± 55	0.46
Large	1592 ± 106	1652 ± 106	60 ± 53	1620 ± 91	1656 ± 101	36 ± 43	0.30
Very large	229 ± 12	243 ± 14	14 ± 8	229 ± 11	238 ± 12	13 ± 7	0.33
Indexes of insulin resistance/sensitivity							
Glucose, mmol/L	5.38 ± 0.10	5.32 ± 0.10	−0.06 ± 0.07	5.40 ± 0.10	5.44 ± 0.14	0.04 ± 0.09	0.34
Insulin, pmol/L	41.3 ± 3.2	39.5 ± 2.9	−1.9 ± 1.9	39.8 ± 3.5	47.3 ± 6.1	7.5 ± 4.5	0.09
HOMA-IR	1.66 ± 0.13	1.55 ± 0.12	−0.11 ± 0.08	1.59 ± 0.14	1.88 ± 0.22	0.29 ± 0.12	0.08
rQUICKI	0.18 ± 0.00	0.19 ± 0.00	0.01 ± 0.00	0.18 ± 0.00	0.18 ± 0.00	0.00 ± 0.00	0.11

1 Values are unadjusted means ± SEMs. *n* *= *54. NEFA, nonesterified fatty acid; rQUICKI, revised quantitative insulin sensitivity index; Δ, change-from-baseline.

2 Linear mixed-model analyses were used to calculate the overall effect of treatment based on Δ values, with adjustments made for fixed effects of baseline values of the assessed variable, period, treatment sequence, gender, age, and BMI. Participant was included as a random effect. The Δ values after each 12-wk dietary intervention were calculated by subtracting week 0 from week 12 values; and week 20 from week 32 values. No period effects were observed in the model for any outcome measure. *P *≤ 0.01 was deemed significant. Data not normally distributed were log transformed.

3 LDL particles were classified by their mean diameter size as small (18.7 nm), medium (23 nm), and large (25.5 nm) (28).

4 HDL particles were classified by their mean diameter size as small (8.7 nm), medium (10.9 nm), large (12.1 nm), and very large (14.3 nm) (28).

**TABLE 4:** Vascular outcomes, blood pressure, endothelial and inflammatory biomarkers, and cardiovascular disease risk scores at baseline (week 0/week 20) and postintervention (week 12/week 32), and the Δ after consumption of the modified and control diets^1^

	Modified diet			Control diet			
Assessment	Baseline	Post	Δ	Baseline	Post	Δ	*P* ^2^
Endothelial function							
%FMD	4.42 ± 0.27	4.77 ± 0.28	0.35 ± 0.15	4.68 ± 0.28	4.14 ± 0.30	−0.51 ± 0.15	<0.0001
Preocclusion artery diameter, mm	3.61 ± 0.09	3.68 ± 0.09	0.06 ± 0.04	3.67 ± 0.10	3.63 ± 0.09	−0.04 ± 0.04	0.08
Peak artery diameter, mm	3.77 ± 0.09	3.85 ± 0.09	0.07 ± 0.04	3.84 ± 0.10	3.77 ± 0.10	−0.07 ± 0.04	0.02
Time to peak diameter, s	53.8 ± 1.9	53.3 ± 2.0	−0.5 ± 1.9	53.7 ± 2.1	58.5 ± 2.2	4.5 ± 2.4	0.04
Arterial stiffness							
PWA-derived AI at 75 beats/min, %	18.9 ± 1.6	19.1 ± 1.4	0.2 ± 0.7	17.1 ± 1.4	18.5 ± 1.4	1.4 ± 0.8	0.62
PWV, m/s	7.6 ± 0.3	7.4 ± 0.2	−0.2 ± 0.5	7.5 ± 0.2	7.8 ± 0.3	0.3 ± 0.4	0.21
DVP-derived stiffness index, m/s	7.6 ± 0.3	7.8 ± 0.4	0.2 ± 0.4	7.9 ± 0.4	8.2 ± 0.3	0.3 ± 0.3	0.96
DVP-derived reflection index, %	69.1 ± 1.9	70.8 ± 1.9	1.7 ± 1.9	71.2 ± 1.8	70.3 ± 1.6	−0.9 ± 1.3	0.57
Clinic BP, mm Hg							
SBP	120 ± 2	119 ± 1	−1 ± 1	120 ± 2	119 ± 2	−1 ± 1	0.44
DBP	70 ± 1	69 ± 1	−1 ± 1	70 ± 1	70 ± 1	0 ± 1	0.24
Ambulatory BP, mm Hg							
24-h SBP	123 ± 2	124 ± 2	1 ± 1	123 ± 2	124 ± 2	1 ± 1	0.90
24-h DBP	74 ± 1	74 ± 1	0 ± 1	74 ± 1	75 ± 1	1 ± 1	0.60
24-h PP	49 ± 1	50 ± 1	1 ± 1	49 ± 1	49 ± 1	1 ± 1	0.64
Day SBP	127 ± 2	128 ± 2	1 ± 1	127 ± 2	128 ± 2	1 ± 1	0.92
Day DBP	77 ± 1	77 ± 1	0 ± 1	77 ± 1	77 ± 1	0 ± 1	0.51
Day PP	50 ± 1	51 ± 1	1 ± 1	50 ± 1	50 ± 1	0 ± 1	0.72
Night SBP	108 ± 2	110 ± 2	2 ± 2	108 ± 2	107 ± 2	−1 ± 1	0.15
Night DBP	63 ± 1	65 ± 1	2 ± 1	63 ± 1	64 ± 1	1 ± 1	0.29
Night PP	45 ± 1	45 ± 1	0 ± 1	44 ± 1	43 ± 1	−1 ± 1	0.26
cIMT, mm	0.59 ± 0.02	0.59 ± 0.02	0.00 ± 0.00	0.58 ± 0.01	0.59 ± 0.02	0.01 ± 0.01	0.83
Circulating biomarkers of endothelial activation and inflammation							
Nitrite, μmol/L	0.13 ± 0.02	0.15 ± 0.02	0.02 ± 0.01	0.15 ± 0.02	0.11 ± 0.02	−0.03 ± 0.02	0.01
Nitrate, μmol/L	17.74 ± 1.60	17.25 ± 1.29	−0.49 ± 1.49	16.96 ± 1.03	16.63 ± 1.30	−0.33 ± 1.56	0.51
VCAM-1, ng/mL	534.9 ± 29.7	537.9 ± 29.7	3.0 ± 20.7	542.8 ± 28.9	499.9 ± 28.3	−40.1 ± 1.7	0.08
ICAM-1, ng/mL	84.4 ± 6.6	79.2 ± 6.5	−5.1 ± 4.3	80.3 ± 8.6	87.0 ± 8.6	6.7 ± 5.5	0.64
E-selectin, ng/mL	27.0 ± 1.9	25.2 ± 1.7	0.6 ± 0.7	24.3 ± 1.7	25.2 ± 1.9	0.8 ± 0.6	0.84
P-selectin, ng/mL	25.2 ± 1.4	26.6 ± 1.6	1.5 ± 0.7	25.9 ± 1.5	26.1 ± 1.5	0.1 ± 0.7	0.30
C-reactive protein, mg/L	2.03 ± 0.76	1.59 ± 0.32	−0.44 ± 0.5	2.12 ± 0.60	1.75 ± 0.42	−0.37 ± 0.62	0.51

1 Values are unadjusted means ± SEMs. *n = *54 (except for DVP, *n* = 46; FMD, *n* = 50; PWA, *n* = 50; PWV, *n* = 29). AI, augmentation index; BP, blood pressure; cIMT, carotid intima media thickness; DBP, diastolic blood pressure; DVP, digital volume pulse; FMD, flow-mediated dilatation; ICAM-1, intercellular adhesion molecule 1; PP, pulse pressure; PWA, pulse wave analysis; PWV, pulse wave velocity; SBP, systolic blood pressure; VCAM-1, vascular adhesion molecule 1; Δ, change-from-baseline.

2 Linear mixed-model analyses were used to calculate the overall effect of treatment based on Δ values, with adjustments made for fixed effects of baseline values of the assessed variable, period, treatment sequence, gender, age, and BMI. Participant was included as a random effect. The Δ values after each 12-wk dietary intervention were calculated by subtracting week 0 from week 12 values; and week 20 from week 32 values. No period effects were observed in the model for any outcome measure. For the key secondary outcome (%FMD response), *P *< 0.05 was deemed significant. For all other secondary outcomes, *P *≤ 0.01 was deemed significant. Data not normally distributed were log transformed.

### Predictive equations of TC and LDL cholesterol

Using the 5 previously published equations summarized by author and year of publication in Müller et al. (27), greater changes in TC and LDL cholesterol concentrations were predicted than the study results observed (Supplemental Table 3). Relative to baseline values, TC concentrations after the modified diet were predicted to increase by 3.1% (31, 35–37) or 2.7% (38), compared with the observed 2.2%. After the control diet, predicted increases in TC were 6.7% (31, 35, 36), 5.7% (37), and 5.3% (38), compared with the observed 5.5%. Similarly, LDL cholesterol concentrations after the modified diet were predicted to increase by 3.5% (35, 36), 2.3% (37), and 4% (38) relative to baseline, whereas after the control diet, values were predicted to increase by 7.6% (35, 36) 6.4% (37), and 8.2% (38), compared with the observed 0.9% and 5.5% increases after the modified and control diets, respectively.

### Endothelial function, indexes of arterial stiffness, and 24-h ABP

There was a significant treatment effect for the %FMD response, representing the NO-mediated vasodilation, whereby the modified diet resulted in an increase in response from baseline (0.35 ± 0.15%), compared with a decrease after the control diet (−0.51 ± 0.15%; *P* < 0.0001) (Table 4). In addition, peak artery diameter increased in response to the modified diet, whereas it decreased after the control diet (*P *= 0.02). Time to peak diameter was found to decrease after the modified diet, whereas it increased after the control diet (*P *= 0.04). There was no significant treatment effect on the preocclusion artery diameter after the 2 intervention diets, compared with baseline values (Table 4). No differences between the 2 treatments were observed for any of the other vascular measurements including cIMT and arterial stiffness (augmentation index measured by PWA), PWV and DVP (stiffness index and reflection index), and 24-h ABP measurement (Table 4).

### NMR metabolomics

There were no significant differences observed in the concentrations of lipoprotein particles within the LDL or HDL subclasses after the 2 intervention diets and compared with baseline values (Table 3).

## Discussion

To our knowledge this is the first study to report the longer-term effects of partially replacing SFAs with MUFAs in a variety of dairy products (UHT milk, cheddar cheese, and butter, modified by a food chain approach) on the fasting lipid profile, endothelial function, and CVD risk biomarkers, in adults at moderate CVD risk. We observed evidence of a significant beneficial effect of the modified diet in attenuating the rise in serum LDL cholesterol and lowering the LDL cholesterol:HDL cholesterol ratio compared with the control diet. A significant increase in the %FMD response and suggestion of an increase in fasting nitrite (a surrogate marker of endothelial NO production) were also evident after modified compared with the control dairy intake.

Our study findings indicate that a high-fat, high-dairy diet including conventional (control) dairy foods led to an increase in LDL cholesterol concentrations (0.19 ± 0.05 mmol/L), which was attenuated by consumption of FA-modified dairy products (0.03 ± 0.06 mmol/L). Our data are in agreement with a review of previously published human trials investigating the chronic impact of FA-modified butter/dairy products, which indicated a tendency toward a beneficial effect on the fasting cholesterol profile (18). This finding may be associated with the impact of dietary FAs on hepatic LDL-receptor expression and activity (39). However, a previous study suggested that 8-wk replacement of 3–6% of dietary SFAs with MUFAs was associated with higher TAG content and particle size of TAG-rich lipoproteins, which may affect LDL residence time in the circulation (40). As suggested in an in vitro study (41), a MUFA-rich meal led to lower competition between postprandial circulating TAG-rich lipoproteins and LDL for uptake by the LDL-receptor, than an SFA-rich meal. This mechanism may potentially explain higher fasting LDL cholesterol after chronic ingestion of the control than after the modified dairy products. Furthermore, there is increasing evidence suggesting that dietary SFA intake may differentially affect LDL particle subclass distribution, with a greater preponderance of smaller, denser particles recognized as more strongly associated with CVD outcomes than larger particles (42). In our study, there was no significant effect on the concentration, or distribution, of particles within the LDL and HDL subclasses, which may be explained by the differential cardiometabolic health effects of our dairy food sources (4) and the relatively modest differences in SFA and MUFA composition between the intervention products (21).

Using 5 previously published equations (31, 35–38), we predicted greater increases in TC and LDL cholesterol concentrations in response to our high-fat, high-dairy diets than the changes observed in the fasting lipid profile. Although it should be noted that reporting mean values for the observed and the predicted equations is associated with errors and does not provide any conclusive information, postintervention TC concentrations after the 2 diets were similar to those predicted by the equation of Clarke et al. (37), which included a coefficient of change in dietary MUFA. Furthermore, the observed increases in LDL cholesterol after the control diet were also slightly lower than predicted. Differences between the predicted and observed LDL cholesterol response after the modified and control diets may be partly explained by the dairy food matrix (4), because the protein and micronutrient contents of the study products were similar across diets (15, 21). It is of note that our intervention diets included a large quantity of butter, which has been shown to elicit a greater fasting LDL cholesterol response than cheese, due to its lower calcium and dairy matrix content (4–6). Therefore, removing butter as an intervention food may have further attenuated the increase in LDL cholesterol concentrations. Future investigations to explore potential mechanisms associated with the FA-modified dairy products matrix on lipid metabolism are required.

Interestingly, the observed attenuation in LDL cholesterol after modified dairy consumption was found even though the intake of dietary total *trans* fatty acids (TFAs) had increased, compared with intake after the control diet (2.5%TE compared with 1.2%TE) (21). Although it was not possible to adequately discriminate between the participants’ intake of industrial and ruminant TFAs (rTFAs) in our 2 diets, supplementation of the bovine diet led to a greater proportion of rTFAs in the modified dairy products (15). The higher TFA intake following the modified diet exceeded the UK recommendation of 2%TE and the current mean intake in adults (0.5%TE) (3). However, the increased TFA intake following our modified diet did not adversely affect fasting lipid biomarkers. This is in agreement with a meta-analysis of randomized controlled trials (RCTs) which found no relation between rTFA daily intake of ≤4.19%TE and CVD lipid risk biomarkers in healthy adults (43). Until there are more definitive data on any association between rTFAs and health, further studies should consider the supplementation of bovines with plant oils using encapsulated technology to protect against ruminal biohydrogenation, which may help to limit the increase in rTFAs that we observed in our modified milk fat (21).

Progression of endothelial dysfunction mediates an increased CVD risk (19, 20). Our finding of a significant improvement in %FMD response after the modified compared with the control diet agrees with an RCT that reported an increased proportion of SFAs in the diet from butter (50 g/d; 19%TE) over a 3-wk period impaired the FMD response relative to 3 isoenergetic diets, including 1 rich in MUFAs (19%TE) from canola margarine (25 g/d) and almonds (45 g/d) (44). Our results lend support to previous studies which found an improvement in the %FMD response after high-MUFA diets, with the beneficial effects of these olive oil–rich diets on endothelial function being primarily attributed to their oleic acid and/or polyphenol content (45–47). A meta-analysis by Inaba et al. (19) concluded that a 1% higher %FMD response at baseline was associated with a 13% lower risk of CVD events, after a mean 3-y follow-up. Based on the meta-analysis (19), it is estimated that consuming our modified diet for 12 wk would represent a 4.6% reduction in future CVD events.

The regulation of vascular tone and function is mediated in part by NO bioavailability (48). Consumption of a diet high in *cis*-MUFAs for 28 d has been reported to result in higher circulating concentrations of plasma nitrates and nitrites (NO_x_) relative to a SFA-rich diet or a low-fat diet enriched in α-linolenic acid, which may reflect a beneficial impact of the MUFA-rich diet on the activity of endothelial NO synthase (49). In support of these data, we observed an increase in plasma nitrite concentrations after the modified diet. It could be speculated that FA-modified dairy product consumption improved endothelial function, thus stimulating NO synthases from endothelial tissue and increasing plasma nitrite concentrations, as described previously in response to a polyphenol-rich olive oil diet (47).

Strengths of our study include its long-term, double-blind, and randomized design. We employed a food chain approach to reduce SFAs in dairy products, which has the potential to prevent movement of SFAs into other food chain entry points (16, 17) and results in “clean” label products favored by consumers (50). A potential limitation of the current study is that our participants were at increased CVD risk and predominantly white (21), thus reducing the generalizability of our findings across different population groups. The implementation of high-fat, high-dairy diets with differential FA composition meant that participants consumed large quantities of dairy products, which may not reflect habitual dairy and total fat intake (21). Future confirmatory investigations with lower, more representative dairy intakes are warranted. Finally, our study was not powered to detect changes in secondary outcomes; therefore, these outcomes should be interpreted with caution.

Our proof-of-concept study is the first to our knowledge to use an agricultural-based reformulation initiative to investigate the impact of SFA-reduced, MUFA-enriched UHT milk, cheddar cheese, and butter on established and novel CVD risk outcomes and indicated that high daily consumption of these products (∼41 g/d of dairy fat) is suggestive of a beneficial attenuation of the rise in LDL cholesterol concentration observed with conventional dairy products in adults at moderate CVD risk. Our findings also indicate that nitrite concentrations and NO-mediated endothelial-dependent vasodilation improved after the FA-modified diet, with the increase in the %FMD response predicted to reduce the risk of CVD events by 4.6% (19).

## Supplementary Material

nqz344_Supplemental_Tables_1-4_and_Figure_1

## Notes

Supported by UK Medical Research Council grant MR/K020218/1 (to JAL). Arla Foods UK and AAK UK supplied in kind the commercially available control dairy study products and high-oleic sunflower oil, respectively, according to our specification.

The funders of the study had no role in study design, data collection, data analysis, data interpretation, or writing of the manuscript.

Supplemental Tables 1–4 and Supplemental Figure 1 are available from the “Supplementary data” link in the online posting of the article and from the same link in the online table of contents at https://academic.oup.com/ajcn/.

The data (in deidentified form) used in the article will be made available to editors upon request either before or after publication for checking. Data described in the article will be made available upon reasonable request.

Abbreviations used: ABP, ambulatory blood pressure; BP, blood pressure; cIMT, carotid intima media thickness; CVD, cardiovascular disease; DVP, digital volume pulse; FA, fatty acid; FMD, flow-mediated dilatation; ICAM-1, intercellular adhesion molecule 1; IPAQ, International Physical Activity Questionnaire; NEFA, nonesterified fatty acid; PL-FA, plasma phospholipid fatty acid; PWA, pulse wave analysis; PWV, pulse wave velocity; RCT, randomized controlled trial; RESET, REplacement of SaturatEd fat in dairy on Total cholesterol; rQUICKI, revised quantitative insulin sensitivity index; rTFA, ruminant *trans* fatty acid; TAG, triacylglycerol; TC, total cholesterol; TFA, *trans* fatty acid; UHT, ultra-high temperature; VAS, visual analog scale; VCAM-1, vascular adhesion molecule 1; Δ, change-from-baseline; %TE, percentage of total energy intake.
