# Supplementary material for: Reformulation initiative for partial replacement of saturated with unsaturated fats in dairy foods attenuates the increase in LDL cholesterol and improves flow-mediated dilatation compared with conventional dairy: the randomized, controlled REplacement of SaturatEd fat in dairy on Total cholesterol (RESET) study
Source: Am J Clin Nutr. 2020 Feb 5;111(4):739–48. doi: 10.1093/ajcn/nqz344 (PMC7138681; doi:10.1093/ajcn/nqz344)
Supplement: nqz344_Supplemental_Tables_1-4_and_Figure_1 [file nqz344_supplemental_tables_1-4_and_figure_1.docx]

**Reformulation initiative for partial replacement of saturated with unsaturated fats in dairy foods attenuates the increase in LDL-cholesterol and improves flow-mediated dilatation compared with conventional dairy: the randomized, controlled REplacement of SaturatEd fat in dairy on Total cholesterol (RESET) study, Vasilopoulou et al.**

**SUPPLEMENTAL TABLES**

**Supplemental Table 1.** Target FA intakes and reported dietary intake at baseline (week 0/week 20) and the Δ following consumption of the modified and control dairy products for 12-wk in adults at moderate risk of cardiovascular disease^1^

|  | Modified | | |  | Control | | |  |  |
| --- | --- | --- | --- | --- | --- | --- | --- | --- | --- |
|  | Baseline | Δ | T^2^ |  | Baseline | Δ | T^2^ |  | *P^3^* |
| Energy, MJ/d | 8.2 ± 0.3 | 1.0 ± 0.4 |  |  | 8.5 ± 0.4 | 0.5 ± 0.3 |  |  | 0.60 |
| Total fat, %TE | 36.1 ± 0.8 | 5.0 ± 1.1 | 38.0 |  | 36.5 ± 0.8 | 3.0 ± 0.9 | 38.0 |  | 0.03 |
| SFA, %TE | 14.2 ± 0.5 | 2.7 ± 0.6 | 16.0 |  | 13.9 ± 0.5 | 5.2 ± 0.6 | 19.0 |  | <0.001 |
| MUFA, %TE | 11.7 ± 0.4 | 3.6 ± 0.5 | 14.0 |  | 11.9 ± 0.4 | -0.1 ± 0.3 | 11.0 |  | <0.0001 |
| n-6 PUFA, %TE | 3.9 ± 0.2 | -0.5 ± 0.2 |  |  | 4.6 ± 0.2 | -1.3 ± 0.2 |  |  | 0.08 |
| n-3 PUFA, %TE | 0.7 ± 0.0 | -0.1 ± 0.1 |  |  | 0.8 ± 0.1 | -0.2 ± 0.1 |  |  | 0.34 |
| Total PUFA, %TE | 4.6 ± 0.2 | -0.4 ± 0.2 |  |  | 5.8 ± 0.4 | -1.4 ± 0.3 |  |  | 0.14 |
| TFA, %TE | 0.9 ± 0.1 | 1.6 ± 0.1 |  |  | 1.0 ± 0.1 | 0.3 ± 0.1 |  |  | <0.0001 |
| Protein, %TE | 16.9 ± 0.5 | -0.7 ± 0.6 |  |  | 16.3 ± 0.5 | -0.2 ± 0.5 |  |  | 0.80 |
| Carbohydrates, %TE | 46.6 ± 1.3 | -4.4 ± 1.5 |  |  | 46.3 ± 0.9 | -3.0 ± 0.9 |  |  | 0.19 |
| Alcohol, %TE | 2.9 ± 0.5 | -0.2 ± 0.5 |  |  | 3.1 ± 0.5 | 0.0 ± 0.4 |  |  | 0.35 |
| Dietary fiber (AOAC), g/d | 20.2 ± 1.1 | -0.8 ± 1.2 |  |  | 20.4 ± 1.1 | 1.6 ± 0.9 |  |  | 0.03 |
| Sodium, g/d | 2.5 ± 0.2 | -0.6 ± 0.1 |  |  | 2.7 ± 0.2 | -0.5 ± 0.1 |  |  | 0.03 |
|  |  |  |  |  |  |  |  |  |  |

^1^ Values are given as unadjusted means ± SEMs. *n =* 52. Dietary intakes estimated from 4-d weighed dietary records at baseline (week 0) and change-from-baseline (Δ). AOAC, association of analytical communities; %TE, percentage of total energy; TFA, t*rans* fatty acids. Adapted from Markey et al. (21).

^2^ Target FA intakes for the modified and control dietary exchange periods.

^3^ Linear mixed model analyses were used to calculate overall effect of treatment based on Δ values, with adjustments made for fixed effects of baseline values of the assessed variable, period, treatment sequence, gender, age and BMI. Participant was included as a random effect. The Δ values following each 12-wk dietary intervention were calculated by subtracting: wk 0 from wk 12 values and; wk 20 from wk 32 values. No period effects were observed in the model for any outcome measure. *P* ≤ 0.01 deemed as significant.

**Supplemental Table 2.** Plasma phospholipid fatty acids at baseline and the Δ following consumption of the modified and control diets for 12-wk in adults at moderate cardiovascular risk^1^

|  |  |  |  |  |  |  |  |
| --- | --- | --- | --- | --- | --- | --- | --- |
|  | Modified | |  | Control | |  | *P^2^* |
|  | Baseline | Δ |  | Baseline | Δ |  |  |
| Total SFA, mol% | 46.5 ± 0.2 | -0.6 ± 0.2 |  | 46.4 ± 0.2 | 0.0 ± 0.2 |  | 0.006 |
| Total *cis*-MUFA, mol% | 11.2 ± 0.2 | 1.0 ± 0.2 |  | 11.0 ± 0.2 | 0.2 ± 0.2 |  | <0.0001 |
| Total *trans*-MUFA, mol% | 0.10 ± 0.00 | 0.23 ± 0.01 |  | 0.12 ± 0.00 | 0.12 ± 0.00 |  | <0.0001 |
| Total n-3 PUFA, mol% | 5.3 ± 0.2 | -0.5 ± 0.1 |  | 5.4 ± 0.2 | -0.4 ± 0.1 |  | 0.58 |
| Total n-6 PUFA, mol% | 35.8 ± 0.3 | 0.0 ± 0.3 |  | 36.2 ± 0.3 | 0.2 ± 0.3 |  | 0.32 |
|  |  |  |  |  |  |  |  |

^1^ Values are unadjusted means ± SEMs. *n* *=* 54. Adapted from Markey et al. (21). Δ, change-from-baseline.

^2^ Linear mixed model analyses were used to calculate overall effect of treatment based on Δ values, with adjustments made for fixed effects of baseline values of the assessed variable, period, treatment sequence, gender, age and BMI. Participant was included as a random effect. The Δ values following each 12-wk dietary intervention were calculated by subtracting: wk 0 from wk 12 values and; wk 20 from wk 32 values. No period effects were observed in the model for any outcome measure. *P* ≤ 0.01 was deemed as significant.

**Supplemental Table 3.** Predictive equations for estimating changes in total and low-density lipoprotein cholesterol in response to dietary fat manipulations (as a percentage of total energy)^1^

| Predictive equations for estimating change in TC and LDL-C | Estimated change following the modified and control diets |
| --- | --- |
|  |  |
| Keys et al. | Modified: ΔTC = 0.17 mmol/L |
| ΔTC = 0.0621ΔS - 0.0310ΔP | Control: ΔTC = 0.37 mmol/L |
| Hegsted et al. |  |
| ΔTC = 0.0543ΔS - 0.03115ΔP - 0.00318ΔM | Modified: ΔTC = 0.17 mmol/L; ΔLDL-C = 0.13 mmol/L |
| ΔLDL-C = 0.0449ΔS - 0.0198ΔP | Control: ΔTC = 0.37 mmol/L; ΔLDL-C = 0.26 mmol/L |
| Mensink and Katan |  |
| ΔTC = 0.0556ΔS - 0.0031ΔM - 0.015ΔP | Modified: ΔTC = 0.17 mmol/L; ΔLDL-C = 0.13 mmol/L |
| ΔLDL-C = 0.033ΔS - 0.006ΔM - 0.014ΔP | Control: ΔTC = 0.37 mmol/L; ΔLDL-C = 0.26 mmol/L |
| Clarke et al. |  |
| ΔTC = 0.052ΔS - 0.026ΔP + 0.005ΔM | Modified: ΔTC = 0.17 mmol/L; ΔLDL-C = 0.08 mmol/L |
| ΔLDL-C = 0.036ΔS - 0.022ΔP - 0.008ΔM | Control: ΔTC = 0.31 mmol/L; ΔLDL-C = 0.22 mmol/L |
| Howell et al. |  |
| ΔTC = 0.0496ΔS - 0.0233ΔP | Modified: ΔTC = 0.15 mmol/L; ΔLDL-C = 0.14 mmol/L |
| ΔLDL-C = 0.0468ΔS - 0.0128ΔP | Control: ΔTC = 0.29 mmol/L; ΔLDL-C = 0.28 mmol/L |
|  |  |

^1^ Summarized predictive equations according to author and publication year are presented as illustrated in Müller et al. (27), followed by the predictive change in total cholesterol and LDL cholesterol for the modified and control diets. LDL-C, low-density lipoprotein cholesterol; M, monounsaturated fatty acids; P, polyunsaturated fatty acids; S, saturated fatty acids; TC, total cholesterol; Δ, change-from-baseline.

**Supplemental Table 4.** Fasting cholesterol profile and ratios at baseline (week 0/week 20) and post-intervention (week 12/week 32), and the Δ following consumption of the modified and control diets^1^

|  | Modified | | |  | Control | | |  |  |
| --- | --- | --- | --- | --- | --- | --- | --- | --- | --- |
|  | Baseline | Post | Δ |  | Baseline | Post | Δ |  | *P^2^* |
|  |  |  |  |  |  |  |  |  |  |
| TC, mmol/L | 5.54 ± 0.13 | 5.66 ± 0.14 | 0.12 ± 0.07 |  | 5.47 ± 0.12 | 5.77 ± 0.13 | 0.29 ± 0.06 |  | 0.08 |
| LDL-C, mmol/L | 3.47 ± 0.11 | 3.50 ± 0.12 | 0.03 ± 0.06 |  | 3.43 ± 0.10 | 3.62 ± 0.11 | 0.19 ± 0.05 |  | 0.03 |
| HDL-C, mmol/L | 1.51 ± 0.04 | 1.55 ± 0.04 | 0.04 ± 0.02 |  | 1.50 ± 0.04 | 1.58 ± 0.05 | 0.07 ± 0.02 |  | 0.55 |
| LDL-C:HDL-C ratio | 2.39 ± 0.09 | 2.33 ± 0.09 | -0.06 ± 0.04 |  | 2.35 ± 0.09 | 2.40 ± 0.09 | 0.05 ± 0.04 |  | 0.04 |
| TC:HDL-C ratio | 3.79 ± 0.12 | 3.76 ± 0.12 | -0.03 ± 0.05 |  | 3.74 ± 0.11 | 3.78 ± 0.12 | -0.04 ± 0.03 |  | 0.13 |
|  |  |  |  |  |  |  |  |  |  |

^1^ Values are unadjusted means ± SEMs. *n* *=* 54. LDL-C, low-density lipoprotein cholesterol; HDL-C, high-density lipoprotein cholesterol; TC, total cholesterol; Δ, change-from-baseline.

^2^ Linear mixed model analyses were used to calculate overall effect of treatment based on Δvalues, with adjustments made for fixed effects of baseline values of the assessed variable, period, treatment sequence, gender, age and BMI. Participant was included as a random effect. The Δ values following each 12-wk dietary intervention were calculated by subtracting: wk 0 from wk 12 values and; wk 20 from wk 32 values. No period effects were observed in the model for any outcome measure. For the primary outcome measure (cholesterol profile), *P* < 0.05 was deemed significant.

**SUPPLEMENTAL FIGURE**


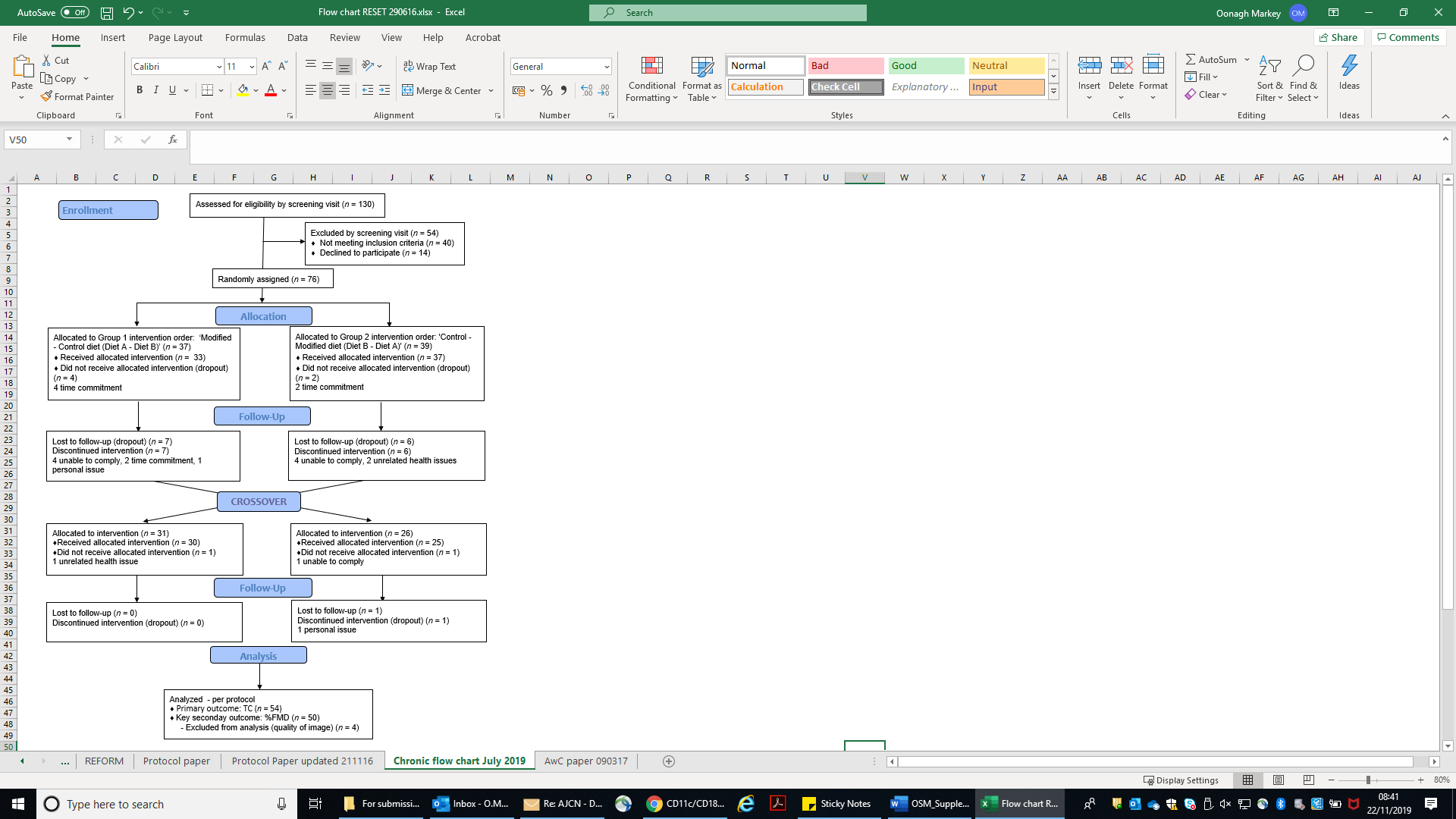


**Supplemental Figure 1.** Flow of participants in the RESET study. % FMD, percentage flow-mediated dilatation response; TC, total cholesterol. Adapted from Markey et al. (21).
